# Convenience Behavior and Being Overweight in Adults: Development and Validation of the Convenience Behavior Questionnaire

**DOI:** 10.3389/fpubh.2019.00020

**Published:** 2019-03-13

**Authors:** Matthias Dreher, Sascha W. Hoffmann, Conny Brendel, David Heser, Perikles Simon

**Affiliations:** ^1^Department of Sports Medicine, Disease Prevention and Rehabilitation, Institute of Sports Science, Johannes Gutenberg University Mainz, Mainz, Germany; ^2^Department of Sports Medicine/Sports Physiology, University of Bayreuth, Bayreuth, Germany

**Keywords:** convenience behaviors, sedentary behaviors, inactivity, physical activity, overweight, obesity, questionnaire

## Abstract

The etiology of overweight and obesity is a mixture of genetic determinants, environmental factors, and health behaviors. Especially intra- and interpersonal inactive behaviors, here termed convenience, seems to play an important role. The objective was to develop and validate the Convenience Behavior Questionnaire (CBQ) to assess convenience-related items and their association with overweight and obesity in a large population. A sample of 1233 subjects aged 18–82 years from six population groups took part in a self-administered questionnaire. Test-retest reliability was estimated and the independent association between convenience-related items and overweight and obesity was investigated. Principal component analysis revealed three factors (avoidance behaviors, social interaction behaviors and domestic environmental factors) which explained 43.4% of the variance contributing to the CBQ. Cronbach's α ranged from 0.80–0.89. Test-retest reliability using intra-class correlation was acceptable ≥ 0.70. Forward stepwise logistic regression analysis, including gender, education level, age and TV viewing on weekends showed a positive relation of convenience behavior and overweight (OR: 1.40; 95% CI: 1.01–1.96; *P* = 0.048), while physical activity status was not significantly associated with overweight (OR: 1.09; 95% CI: 0.77–1.54; *P* = 0.629). The CBQ seems to be a reliable tool which considers non-traditional behaviors related to overweight development. Interestingly our findings revealed a better relationship between convenience-related behavior with overweight and obesity than the habitual physical activity score.

## Introduction

The etiology of overweight and obesity has been attributed to a complex mixture of genetic determinants (1–3), environmental factors (4–6) and unfavorable health behaviors (7–10). These unhealthy behaviors are often described as the presence of sedentary behaviors such as insufficient physical activity, high screen time activity, or risk factors like excessive caloric intake, reduced sleep and smoking (7–15). An astounding 30% of EU citizens stated in 2013 that they are never or seldom physically active more than once a week (16). Furthermore, it may be possible that people meet current physical activity guidelines, but also engage several hours per day in sedentary behaviors and are therefore not physically active (17). This finding supports the relative importance of physical inactivity, sedentary behaviors, energy balance and dietary patterns of the obesity epidemic and its accompanying diseases (18–23).

A large amount of literature reports that certain personality traits are related to lifestyles which promote health (24–26). In particular, people who scored higher on extraversion, conscientiousness, and openness to experience were more likely to engage in a variety of activity types of behavior (27, 28), while overweight and obese individuals scored lower on conscientiousness and openness to experience (29, 30).

However, Jokela et al. emphasized that only conscientiousness reflecting high self-control, orderliness, and adherence to social norms, may be a robust, broad-level personality trait associated with reduced obesity risk (31). This would imply that specific intra- and interpersonal inactive behaviors, here termed convenience, could be factors associated with being overweight and with obesity independent from a physically active lifestyle. Also, it should be taken into account that several of the convenience behaviors could be passive, while others would be done with deliberate intent and obvious volition (32).

Some personality traits also imply a relevant association for avoidance behaviors, social behaviors or behaviors related to the domestic environment with obesity and being overweight. The knowledge from the theoretical considerations indicates that avoidance behavior is i.e., a self-protective coping strategy and creates barriers to a healthy lifestyle and a healthy body weight (33–36). The individual social context (i.e., social networks, new media usage, social support) influences health behaviors and behavior changes which may lead to negative changes in eating behavior, physical activity, and body weight (37–41). Within a domestic environment (i.e., housework, having less cooking skills, doing fewer chores or convenience orientation toward ready meal preparation) has an influence on weight and therefore, on the overweight status (42–44).

These factors are not yet considered systematically in a uniform evaluation tool and seem to be underinvestigated or not deemed relevant in the current public health debate. To the best of our knowledge, we present the first study which investigates the possibility to assess items reflecting convenience behavior in a broader sense by using a score value derived from a standardized questionnaire. For this purpose, we derived health behavior connected questions from the dimensions “avoidance,” “social,” and “domestic” and set them into relation of the domains of “work,” “leisure-time,” “domestic and family” which are shown to a have a overweight and obesity relation.

These domains were chosen to achieve congruence in structure and evaluation to the Habitual Physical Activity Questionnaire (HPAQ) of Baecke et al. which differentiates between physical activity at work, physical activity during leisure time and sports during leisure time (45).

The development of an appropriate, valid and reliable assessment tool in the field of overweight prevention is necessary to better understand if the convenience behavior in daily life is truly independent of the known influencing factors such as habitual physical activity (HPA). The present study (a) investigates the construct validity of a self-administered questionnaire about convenience-related behavior, to establish meaningful indices of convenience, (b) estimates the test-retest reliability, and (c) investigates the independent association between convenience-related items and overweight and obesity status in a large adult population.

## Materials and Methods

### Development of the Convenience Behavior Questionnaire (CBQ)

The aim of the questionnaire is to measure terms of convenience behaviors in three domains analogous to the HPAQ of Baecke et al. (45) in terms of physical activity. The HPAQ is easy to administer and a short, practical assessment tool to extrapolate a general more long-lasting physical activity level (45). The HPAQ has furthermore been evaluated and validated in previous research studies (46) as well as with obese individuals (47, 48).

The knowledge from the theoretical considerations, that avoidance behavior (33–36), social related behaviors (37–41) and daily behaviors around the domestic environment (42–44) seem to influence weight and therefore, the overweight status, sets scientific foundation for the questionnaire development. In total 24 items pertaining to the domains of work environment, during leisure-time, in private life and in the domestic environment were derived and connected, to the three behavior dimensions.

The developmental process included: Initial questionnaire development, refinements based on focus group responses, refinements based on responses from researchers, pilot test using a small sample of faculty staff at a public university, and validation using a heterogeneous study sample. A five-point Likert-scale was used to determine the level of agreement with the questions by answering “fully agree” to “do not agree.”

### Population and Procedure

In order to analyze the pattern of factors, we decided to distribute our questionnaire to a collective that was supposedly rather heterogeneous regarding the potential convenience.

Since we could not yet estimate any differences between groups or variances in the sub-items of the questionnaire, a concrete calculation for the estimation of case numbers was not possible. Therefore, we decided to restrict ourselves to a size of the collective that is in the typical range for studies that intend to implement a novel survey instrument, such as the Baecke Score (45).

The sample of participants consists of different population groups with a large number of occupations, different daily behaviors, activity patterns, educational- and socioeconomic backgrounds to provide a widespread but possibly representative sample (49, 50). The following six population groups were selected: Kindergarten teachers and primary school teachers, bus drivers, members of church choirs, and parents of school children and parents of kindergartners.

Kindergarten teachers and primary school teachers were selected because kindergarten teachers seem to have an increased prevalence of being overweight and obese than other job types in Germany (51). Due to an increased risk of childhood overweight between the ages of 5–8 years old (52), primary school teachers were included, because they are important role models for physical activity (53). The parents of school children and parents of kindergartners were recruited due to the diversified range of occupations without a preselection and the highest influence on children's health behaviors besides kindergarten teachers (54). Additionally, two extreme groups were included to extend the data collection. Bus drivers have a low physical activity level and a limited decision space during work time. A long tenure as a driver may lead to sedentary behavior, lack of exercise, and unhealthy diet which increases the risk of obesity (55). Morris and Raffle already showed in 1954 that bus drivers have a higher risk for coronary heart disease compared to conductors and discussed the role of physical activity (56). Church choir members have been described as dedicated and loyal people with a regular commitment to attend rehearsal which motivates them to avoid physical inactivity and provides a sense of balance to other pressures in life (57).

All individuals were informed in advance about the aims of the study by email and further methods of recruitment had to be used to generate a high response rate within the described population groups. Parents of school children and kindergartners as well as primary school teachers were informed during informative meetings in the respective school or kindergarten. The bus drivers were informed and questioned prior to additional staff meetings and members of church choirs were visited and informed during their weekly choir practice. After participants had completed the questionnaire, they were pleased to drop the filled-in questionnaire into a box to ensure responder anonymity. Participants were asked about age, gender, height, weight, education level, screen time activity, HPAQ, and the 24 questions to develop the CBQ.

The BMI of the participants was calculated from self-reported height and weight and classified into underweight (BMI < 18.5 kg/m^2^), normal weight (BMI = 18.5–24.9 kg/m^2^), overweight (BMI = 25.0–29.9 kg/m^2^), and obese (BMI ≥ 30.0 kg/m^2^) (58).

Education level was used as a proxy for the individual socioeconomic status and was categorized into three levels: Low educational level (no education, to have an apprenticeship or secondary modern school), middle educational level (vocational- and technical school or secondary school), high educational level (university degree or technical college degree) (59).

Screen time activity was assessed by questions about the duration of television (TV) viewing, digital versatile disc (DVD) viewing and the duration of computer use including internet use, using a five-point Likert-scale (1 = never, 2 = ~30 min per day, 3 = 1–2 h per day, 4 = 3–4 h per day, 5 = more than 4 h per day) (60). Additionally, it has differed between screen time activity on weekdays and weekends.

For a test-retest study, parents of 5.5–6 year-old (*n* = 139) pre-school children were pleased to give their written consent to participate in an epidemiological longitudinal study. Approximately 10 months after the first examination 23 persons of the sub-sample gave their consent to participate again.

### Statistical Analysis

The results section consists of two parts. The first part describes the basic descriptive characteristics, body mass index, screen time activities and physical activity scores of the participants. The second part focuses on the development of the convenience score by identifying meaningful dimensions of convenience behaviors. A frequent approach to determine relevant variables from questionnaires is based on statistical exploratory methods.

One method which is predominantly used in the context of physical activity behavior and dietary patterns as well as in the research field of diagnostics of depression is principal component analysis (PCA) to provide evidence of a stable factor structure (45, 61, 62). PCA aims to classify variable information into a few factors by analyzing the covariance structure.

Items with factor loadings > 0.4 were considered as significantly contributing to a factor. A good correspondence between valid and sampling factor structure occurs when factor stability is ≥ 0.9 (63). Factor stability was applied also following the recommendations of Guadagnoli and Velicer (63). Analysis of eigenvalues in the screen plot and the commonly applied eigenvalue criterion (> 1.0) were used to determine the number of factors remaining for the final CBQ-scale (64).

Cronbach's α was used to evaluate internal consistency (65, 66). Cronbach's α is considered to be acceptable for values > 0.6 (67). The test-retest reliability of the factors was assessed using intra-class correlation (ICC). ICC estimates above 0.75 were considered as good reliability scores, estimates between 0.50 and 0.75 as moderate reliability scores, and estimates below 0.50 as poor reliability scores, respectively (68).

Kruskal-Wallis-ANOVA and one-way ANOVA with *post hoc* test (Bonferroni) were used to evaluate differences in the convenience-related items from the six population groups: A: Kindergarten teachers; B: Bus drivers; C: Members of church choirs; D: Parents of school children; D: Primary school teachers; F: Parents of kindergartners.

Initial χ^2^ analyses prove significant relation of the covariates and the CBQ, whereas additional forward stepwise logistic regression analysis was used to create the questionnaire (model) and prove the association between overweight and obesity and the CBQ, age, gender, education level, screen time activity and habitual physical activity. Continuous variables were transferred to categorical variables by median split. χ^2^ tests with contingency tables were employed to test if the items of the HPAQ and CBQ were statistically independent of one another.

The Alpha-Level was set a priori at *P* ≤ 0.05. Statistical analyses were calculated with SPSS PASW 22 Statistics (IBM Corp., Somers, NY) and JMP 8.0 (SAS, Cary, NC).

## Results

### Demographics

Table 1 summarizes the basic characteristics of the 1233 participants. Participants were from different socio-economic status (low-, middle-, and high educational level).

**Table 1:** Demographic characteristics of the 1233 participants of the Convenience Behavior Questionnaire (CBQ; *n* = 1233).

**Variables**	***n***	**%**	**Mean ± SD^**a**^**	**Range**
**GENDER**				
Female	950	77.0	-	-
Male	283	23.0		
**LIFE STAGE (AGE)**				
18-30	182	14.8	40.0 ± 10.1	18.0–82.0
31-40	544	44.1		
≥41	507	41.1		
**SOCIO-ECONOMIC STATUS**				
Low educational level	337	27.3	-	-
Middle educational level	428	34.7		
High educational level	468	38.0		
**BMI**				
Underweight	31	2.5	24.8 ± 4.5	16.1–48.1
Normal weight	695	56.4		
Overweight	336	27.3		
Obese	171	13.9		
**SCREEN VIEWING ACTIVITY**				
TV/DVD viewing on weekdays	656	53.2	-	-
<3 h/day	565	86.1		
≥3 h/day	91	13.9		
TV/DVD viewing on weekends	657	53.3	-	-
<3 h/day	453	68.9		
≥3 h/day	204	31.1		
Computer use on weekdays	658	53.4	-	-
<3 h/day	543	82.5		
≥3 h/day	115	17.5		
Computer use on weekends	658	53.4	-	-
<3 h/day	580	88.1		
≥3 h/day	78	11.9		
**HABITUAL PHYSICAL ACTIVITY (HPA)**^**b**^				
Work index	-	-	2.6 ± 0.6	1.3–4.6
Sport index			2.6 ± 0.6	1.0–7.5
Leisure-time index			3.1 ± 0.7	1.3–5.0
HPA score			8.3 ± 1.2	4.8–14.4

a *SD = standard deviation*.

b *Sign. gender differences in the indices of physical activity (P ≤ 0.05)*.

The majority of all participants were primarily women (77.0%). The sample age ranged from 18–82 years (Mean ± SD; 40.0 ± 10.1). BMI ranged from 16.1–48.1 (24.8 ± 4.5). A normal BMI was stated for 56.4% (695) of the study population, followed by 336 (27.3%) overweight participants. A BMI > 30.0 was classified as obese which pertains to 171 persons (13.9%). A small fraction of 31 participants (2.5%) were underweight.

The mean score of HPAQ (8.3 ± 1.2) and the three partitioned sub-index Work index (Mean 2.6 SD ± 0.6), Sport index (Mean 2.6 SD ± 0.6) and Leisure-time index (Mean 3.1 SD ± 0.7) were analogous with the values in the original publication (45).

### Principal Component Analysis

Using principal component analysis and varimax-rotation as the extraction method, scree-plots revealed three factors that accounted for 43.4% of the variance. Kaiser-Meyer-Olkin criterion was acceptable (0.771) and factor stability was excellent (0.991). The varimax-rotated three-factor solution extracted items which had similar loadings on the same factor and could be combined into one factor. Answers regarding the same questions in the working context and in the leisure time context were significantly correlated with each other. Spearman rank correlation coefficients for all factors were between (*r* = 0.501–0.628; *P* < 0.01). Items that describe social interaction behavior and avoidance behavior during working time did not contribute as significantly to explain the variance of the convenience index, as the respective items during leisure time. Therefore, only the leisure time items remained in the final index, since they were more relevant for explaining the variance. Finally, nine of the original 24 items were excluded.

Table 2 shows the factor-loading matrix of the 15 remaining items from the final extraction method. Data on males and females were pooled because of similar patterns in the gender-specific analysis.

**Table 2:** Factor-loading matrix and Cronbach's α of the items about convenience-related behavior (*n* = 1233).

**Factor and question number**		Factor loadings^*^
**FACTOR 1: AVOIDANCE BEHAVIOR INDEX (ABI)**^**a**^		
1.	In order to achieve my personal goals, I always choose the easiest possible way.	0.735
2.	If I compare myself with age peers, I am generally “more convenient” than others.	0.729
3.	I avoid private disputes/conflicts.	0.719
4.	If it is possible for me, I avoid physical activity in everyday life.	0.704
**FACTOR 2: SOCIAL INTERACTION BEHAVIOR INDEX (SIBI)**^**b**^		
5.	If it is possible, I try to personally meet persons in my leisure-time for social networking.	0.715
6.	I'm looking forward to learn something new and/or to gain new experiences.	0.684
7.	I use new media in order to save time.	0.621
8.	I actively contact with other age peers, to improve myself and my personal performance.	0.552
9.	In social contexts, I would make decisions, even if disadvantages arise from it.	0.431
**FACTOR 3: DOMESTIC ENVIRONMENT INDEX (DEI)**^**c**^		
10.	When I cook, I use mostly fresh ingredients and less finished products.	0.717
11.	I care about information about the food security and quality.	0.673
12.	I support social projects/groups and work as a volunteer, respectively.	0.517
13.	Even on vacation I like to get up early to seize the day.	0.506
14.	Domestic score	0.474
15.	I make sure that my everyday environment is always neat/tidy.	0.453

* *Factor loadings < 0.4 were supressed*.

a *Cronbach's α = 0.801*.

b *Cronbach's α = 0.891*.

c *Cronbach's α = 0.854*.

The first factor of the 3-factor-solution consisted of four items with high loadings and accounted for 20.0% of the variance in the explained model (eigenvalue = 3.0). Because these items were referring to avoidance behaviors, this factor was named “Avoidance Behavior Index” (ABI).

The second factor consisted of five items and accounted for 14.8% of the variance (eigenvalue = 2.2). This factor is indicative of daily behavior patterns during social interaction context which is based on individual initiative and was labeled “Social Interaction Behavior Index” (SIBI).

The third factor consisted of six items and accounted for 8.6% of the variance in the explained model (eigenvalue = 1.3). This factor is indicative of daily behavior patterns in the domestic environment and named “Domestic Environment Index” (DEI).

### Assessment of Internal Consistency and Reliability

Internal consistency for the CBQ was assessed by Cronbach's α. All outcomes were acceptable (i.e., ≥ 0.7) as follows: ABI (α = 0.801), SIBI (α = 0.891), and DEI (α = 0.854) (Table 2). The participants of the test-retest study (n = 23), and the test-retest reliability for the ABI (ICC = 0.75) and the SIBI (ICC = 0.65) were good and moderate, whereas the DEI (ICC = 0.53) and the total Convenience Behavior Index (CBI, ICC = 0.62) provide moderate test-retest reliability (68), respectively. Noteworthy, all items related to a non-convenient dimension, like most questions in the dimension of the DEI and the SIBI were recoded into the opposite dimension.

Table 3 presents the mean scores of convenience-related items in males and females. Total CBI score (male mean 9.8 SD ± 1.1; female mean 10.5 SD ± 1.1) as well as the ABI, SIBI, and DEI, mean scores were significantly higher in females than in males. Each factor was computed to derive a total score between 1 and 5. The lower the score in the CBI, the more likely the participants had daily convenience-related behavior patterns.

**Table 3:** Mean scores of convenience-related behavior indices in males and females (*n* = 1233).

**Index**	**Males (*****n*** **=** **283)**		**Females (*****n*** **=** **950)**		*P*-value^*^
	**Mean**	***SD***	**Mean**	***SD***	
Avoidance behavior index	3.5	0.8	3.9	0.7	<0.001
Social interaction behavior index	3.2	0.5	3.3	0.5	<0.001
Domestic environment index	3.1	0.6	3.3	0.6	<0.001
Convenience behavior index	9.8	1.1	10.5	1.1	<0.001

* *Mann-Whitney-U-Test; independent t-test for gender differences of the CBI factors*.

The descriptive characteristics of convenience-related items between different clusters in males and females are described in Table 4. Highest ABI for females was found in the primary school teachers (Mean 4.1 SD ± 0.5) while the lowest score was found in church choirs (Mean 3.4 SD ± 0.8). Focusing on female SIBI the bus drivers showed the highest score (Mean 3.7 SD ± 0.6) and lowest for mothers of school children (Mean 3.2 SD ± 0.5). Female DEI related convenience was as well highest in bus drivers (Mean 3.7 SD ± 0.9) and lowest in kindergarten teachers (Mean 3.2 SD ± 0.5).

**Table 4:** Descriptive characteristics of convenience-related items between different clusters^†^ in males and females (*n* = 1233).

**Gender**	**Index (Mean ±*SD*)**	**Cluster**^**†**^						
		**A**	**B**	**C**	**D**	**E**	**F**	*P*-value^*^
**Females (*n* = 950)**		**(*n* = 177)**	**(*n* = 3)**	**(*n* = 54)**	**(*n* = 270)**	**(*n* = 48)**	**(*n* = 398)**	
	Avoidance behavior index^a^	4.0 ± 0.5	4.0 ± 0.9	3.4 ± 0.8	3.9 ± 0.7	4.1 ± 0.5	3.8 ± 0.6	<0.001
	Social interaction behavior index^b^	3.5 ± 0.5	3.7 ± 0.6	3.4 ± 0.5	3.2 ± 0.5	3.4 ± 0.5	3.3 ± 0.5	<0.001
	Domestic environemt index	3.2 ± 0.5	3.7 ± 0.9	3.4 ± 0.7	3.4 ± 0.6	3.3 ± 0.6	3.3 ± 0.6	0.104
	Convenience behavior index^c^	10.8 ± 0.9	11.4 ± 2.2	10.1 ± 1.4	10.5 ± 1.2	10.9 ± 1.2	10.5 ± 1.1	0.001
**Males (*****n*** **=** **283)**		**(*****n*** **=** **15)**	**(*****n*** **=** **97)**	**(*****n*** **=** **46)**	**(*****n*** **=** **50)**	**(*****n*** **=** **7)**	**(*****n*** **=** **68)**	
	Avoidance behavior index^d^	3.9 ± 0.6	3.4 ± 0.8	3.2 ± 0.8	3.7 ± 0.7	3.8 ± 0.7	3.8 ± 0.6	<0.001
	Social interaction behavior index^e^	3.1 ± 0.5	3.3 ± 0.5	3.3 ± 0.5	3.1 ± 0.5	3.2 ± 0.4	3.2 ± 0.5	0.049
	Domestic environment index^f^	2.7 ± 0.5	3.0 ± 0.6	3.1 ± 0.7	3.1 ± 0.6	3.1 ± 0.3	3.3 ± 0.6	0.026
	Convenience behavior index^g^	9.6 ± 1.2	9.8 ± 0.9	9.5 ± 1.5	9.8 ± 1.2	10.0 ± 1.1	10.3 ± 1.0	0.010

* *Kruskal-Wallis ANOVA; univariate ANOVA (Bonferroni) of convenience-related behavior items between the different population groups*.

† *Definition of clusters:*

**Table:** 

A	Kindergarten teachers
B	Bus drivers
C	Members of choirs
D	Parents of school children
E	Primary school teachers
F	Parents of kindergartners

a *Multiple comparisons*. *sign. at P < 0.05 between Cluster C and Cluster A, D, E, F; between Cluster A and Cluster F; and between Cluster F and Cluster D, E*.

b *Multiple comparisons sign. at P < 0.05 between Cluster A and Cluster D, F*.

c *Bonferroni post-hoc test sign. at P < 0.05 between Cluster A and Cluster C; and between Cluster C and Cluster E*.

d *Multiple comparisons sign. at P < 0.05 between Cluster B and Cluster F; and between Cluster C and Cluster D, F*.

e *Multiple comparisons sign. at P < 0.05 between Cluster B and Cluster D*.

f *Multiple comparisons sign. at P < 0.05 between Cluster A and Cluster F*.

g *Bonferroni post-hoc test sign. at P < 0.05 between Cluster C and Cluster F*.

Male kindergarten teachers (Mean 3.9 SD ± 0.6) had the highest ABI, whereas in consistence with the females the male church choir members had the lowest score for ABI (Mean 3.2 SD ± 0.8). Just a very low difference was found in SIBI score. The group of male kindergarten teachers (Mean 3.1 SD ± 0.5) and fathers of school children (Mean 3.1 SD ± 0.5) showed lowest SIBI scores, whereas highest scores were shown in bus drivers (Mean 3.3 SD ± 0.5) and church choir members (Mean 3.3 SD ± 0.5). Fathers of kindergarten children show the highest value (Mean 3.3 SD ± 0.6) in the DEI and accompanied with females, the lowest score was found in kindergarten teachers (Mean 2.7 SD ± 0.5). Female (Mean 10.1 SD ± 1.4) and male (Mean 9.5 SD ± 1.5) members of church choirs had the lowest mean scores on the total CBI score, whereas the highest values were found in female bus drivers (Mean 11.4 SD ± 2.2) and the multiple jobs containing a group of male parents of kindergarten children (Mean 10.3 SD ± 1.0).

### Analysis of Variance (ANOVA)

The results of Kruskal-Wallis ANOVA and one-way ANOVA with *post-hoc* comparisons (Bonferroni) were listed in Table 4. Significant gender differences between convenience-related scores and the six different population groups were considered except for mean scores of the DEI between the different population groups in males.

### Variables Associated With Overweight and Obesity

χ^2^ analyses indicated that gender, education level, age, population group, TV viewing on weekends, CBI score and ABI were significantly related to being overweight and obesity status (data not shown), whereas TV viewing on weekdays, SIBI (OR: 0.91; 95% CI: 0.73–1.15; *P* = 0.452); DEI (OR: 0.84; 95% CI: 0.67–1.06; *P* = 0.148) and the HPA score (OR: 1.09; 95% CI: 0.77–1.54; *P* = 0.629) were found to have no significant association with overweight and obesity status.

Subsequently, a forward stepwise logistic regression was performed to determine variables which are suited to predict overweight and obesity. Omnibus tests showed model significance at *P* < 0.001. Estimated odds ratio (OR) with the 95% confidence interval (CI) is shown in Table 5. Female individuals were less likely to be overweight or obese as compared with male participants (OR: 0.44; 95% CI: 0.27–0.71). Participants with low educational level were 3.01 times (95% CI: 1.94–4.67) more likely to be overweight or obese than participants with a high educational level. A positive prediction was found for individuals watching TV on weekends equal or more than 3 h per day as well. There is a 1.57-fold increased risk (95% CI: 1.09–1.96) to be overweight or obese compared to individuals watching TV less than 3 h per day. The total CBQ did not remain in the model when analyzed with ABI. In addition, ABI showed an association between being overweight and obesity status. Participants with a low ABI score and therefore a higher daily convenience behaviors were 1.40 (95% CI: 1.01–1.96) times more likely to be overweight or obese as compared with active participants in the high scoring group (4.00–5.00).

**Table 5:** Relations between demographic characteristics^a^ and the Avoidance Behavior Index (ABI) predicting overweight and obesity (*n* = 1233).

**Variables**	**OR^**b**^**	**95% CI^**c**^ for OR**	*P*-value^*^
**GENDER**			
Female	0.44	0.27–0.71	0.001
Male	1.00		
**SOCIO-ECONOMIC STATUS**			
Low educational level	3.01	1.94–4.67	<0.001
Middle educational level	1.48	0.98–2.22	0.060
High educational level	1.00		
**TV VIEWING ON WEEKENDS**			
≥3 h/day	1.57	1.09-1.96	0.016
<3 h/day	1.00		
**AVOIDANCE BEHAVIOR INDEX**^**d**^			
1.00–3.75	1.40	1.01–1.96	0.048
4.00–5.00	1.00		

a *Results of a forward stepwise logistic regression model. Only sign. associations are displayed*.

b *OR, Odds ratio*.

c *OR and 95% confidence interval are from forward regression analyses in which all independent variables listed were included in the model simultaneously*.

d *Avoidance Behavior Index scores ranged from 1.00 to 5.00. Scores were dichotomized by median split. The lower the score, the more likely the participant had daily convenience behavior patterns*.

* *P ≤ 0.05*.

The final Convenience Behavior Questionnaire (CBQ) calculation guideline of the remaining 15 questions, including the three different sub-scores (Avoidance Behavior Index (ABI), Social Interaction Behavior Index (SIBI) and Domestic Index (DEI) are shown in Table 6.

**Table 6:** Questions, codes, and method of calculation of indices of the Convenience Behavior Questionnaire (CBQ).

**1**	**In order to achieve my personal goals, I always choose the easiest possible way**. fully agree/mostly agree/agree/seldom agree/do not agree		**1–2–3–4–5**
**2**	**If I compare myself with age peers, I am generally “more convenient” than others**. fully agree/mostly agree/agree/seldom agree/do not agree		**1–2–3–4–5**
**3**	**I avoid private disputes/conflicts**. fully agree/mostly agree/agree/seldom agree/do not agree		**1–2–3–4–5**
**4**	**If it is possible for me, I avoid physical activity in everyday life**. fully agree/mostly agree/agree/seldom agree/do not agree		**1–2–3–4–5**
**5**	**If it is possible, I try to personally meet persons in my leisure-time for social networking**. fully agree/mostly agree/agree/seldom agree/do not agree		**1–2–3–4–5**
**6**	**I'm looking forward to learn something new and/or to gain new experiences**. fully agree/mostly agree/agree/seldom agree/do not agree		**1–2–3–4–5**
**7**	**I use new media in order to save time**. fully agree/mostly agree/agree/seldom agree/do not agree		**1–2–3–4–5**
**8**	**I actively contact with other age peers, to improve myself and my personal performance**. fully agree/mostly agree/agree/seldom agree/do not agree		**1–2–3–4–5**
**9**	**In social contexts, I make decisions, even disadvantages arise from it**. fully agree/mostly agree/agree/seldom agree/do not agree		**1–2–3–4–5**
**10**	**When I cook, I use mostly fresh ingredients and less finished products**. fully agree/mostly agree/agree/seldom agree/do not agree		**1–2–3–4–5**
**11**	**I care about information about the food security and quality**. fully agree/mostly agree/agree/seldom agree/do not agree		**1–2–3–4–5**
**12**	**I support social projects/groups and work as a volunteer, respectively**. fully agree/mostly agree/agree/seldom agree/do not agree		**1–2–3–4–5**
**13**	**Even on vacation I like to get up early to seize the day**. fully agree/mostly agree/agree/seldom agree/do not agree		**1–2–3–4–5**
**14**	**How many people live in your household?**	1-2/3-4/≥5	**0.76–1.26–1.76**
	**How much time do you need to do the chores (cooking, washing, cleaning etc.)? [h/week]**	<10/10-20/20-30/30-40/>40 Proportion (1 year)	**0.5–1.5–2.5–3.5–4.5** **0.92**
	**You live in an apartment/ a house?**	**Apartment/house**	**0.76–1.76**
	**How much time do you need to do the housework (craft activities, detail work)? [h/week]**	<1/1-3/4-6/7-9/>9 Proportion (1year)	**0.5–1.5–2.5–3.5–4.5** **0.92**
**15**	**I make sure that my everyday environment is always neat / tidy**. fully agree/mostly agree/agree/seldom agree/do not agree		**1–2–3–4–5**
**Calculation of the domestic score (DS; I 14):** (a score of zero is given to people who reach a minimal score-value and currently have to do less domestic work [i.e. single household]) DS = (14a ^*^ 14b ^*^ proportion) + (14c ^*^ 14d ^*^ proportion) − 0.6992 **= 0/0.01 – <4/4 – <8/8 – <12/>12**			**1–2–3–4–5**

*Calculation of scores of the indices of the CBQ*:

Avoidance Behavior Index (ABI) = (I_1_ + I_2_ + I_3_ + I_4_)/4; Social Interaction Behavior Index (SIBI) = ([6-I_5_] + [6-I_6_] + I_7_ + [6-I_8_] + [6-I_9_])/5;

*Domestic Environment Index (DEI) = ([6-I_10_] + [6-I_11_] + [6-I_12_] + [6-I_13_] + I_14_ + [6-I_15_])/6*.

## Discussion

This research was a first attempt to develop and test a brief and short questionnaire for measuring convenience behaviors and to examine its relationship with overweight and obesity in adults. The study offers a model which considers non-traditional behavioral factors which are involved in the obesity etiology. Different factors were summarized into three subcategories in the final CBQ and revealed patterns of daily convenience: Avoidance Behavior, Social Interaction Behavior, and behavior in the Domestic Environment.

Four questions (items 1–4) identified avoidance behaviors to reduce inconvenient circumstances in the personal environment, including questions about avoiding “disputes/conflicts” or “the intention to be physically active,” as well as the self-assessment about being convenient comparing to other same age peers. Additionally, items 1–4 had the highest loadings in the PCA and resulted in 20.0% of the explained variance. The results of the logistic regression analysis presume that the ABI is an independent predictor of overweight status in adults. These findings are in accordance with results from Latner who examined avoidance behaviors in obese weight-loss participants. Latner indicated in women, that greater avoidance behaviors were associated with lower success at achieving personal weight-loss goals (69). Morrison et al. reported that avoidance behaviors, as an idiom of distress, were more present in overweight and obese adolescent boys (36).

Individuals who reported that they tend to not finish tasks or give up easily may more often persist in efforts to regulating addictive eating behaviors which might lead to higher BMI values (70). Therefore, a further explanation approach is that ABI could possibly explain the recently observed indirect association between low levels of task persistence (lack of perseverance) and BMI as a function of food addiction symptoms (25, 70). In addition, BMI and waist circumference were significantly increased in individuals who were less dutiful (30).

The five questions inquiring on social interaction behaviors integrated items about the willingness for social interaction and being proactive to “personally meet persons in real life for social networking” or “the intrinsic motivation to gain new experiences” as well as the knowledge about making “decisions, even disadvantages arise from it” and the “use of new media.” Males have significantly lower score values in the SIBI compared to females. We observed no statistically significant association between social interaction behaviors and overweight and obesity.

The study participants were average in weight rather than overweight if they show convenience in maintaining their social contexts (i.e., social environment or social network). The social related behaviors which were observed in our study are consistent with research findings of Patel and Schlundt, who pointed out that eating in a social context is an independent risk factor of increased food intake (71). Results from the study of Hetherington et al. show that eating with friends and acquaintances or while TV viewing stimulated greater food intake than when eating alone or with strangers (72). Koehly et al. suggests that social relationships may contribute to the development of obesity through the interaction of behavioral and environmental factors and furthermore, a social network approach can strengthen health prevention interventions already in adolescence (38). Lindsay et al. reported, that the feelings of isolation and the lack of help from friends or family are important changes affecting their lives and limited their ability to consume healthy meals (39). Different studies provide evidence that openness to experience significantly contributes to social function throughout life (28). Time-saving opportunities of new media use were already shown in 2002 by Alreck and Settle. New media use like online shopping seems to be more time saving than traditional modes of shopping. Furthermore, a positive relation was found for hours worked outside the home, with online shopping and general new activity (i.e., online banking) (73). It can be suggested that the time saved by new media use is useful for social interaction behaviors.

In summary, social networks may play an important role in overweight and obesity development with positive or even negative effects (41). Already it younger age, individual social contexts appear to influence health and therefore may be important in treatment and prevention programs for being overweight and obese (74).

Questions 10–15 inquire about the domestic environment i.e., supporting “social projects/groups,” “behaviors on vacation,” questions about “food security and quality” and the use of “mostly fresh ingredients and less convenience.” In accordance to literature, a domestic environment score was calculated from a combination of how many people live in the household, the home environments (apartment or house) the amount of time per week doing the chores, cooking, washing, etc. and the housework (craft activities etc.) (42, 43). For the method of calculation see Table 6.

No significant association between items of the DEI and the risk of being overweight or obese was found. A reason might be that overweight individuals may self-report domestic tasks more intensely than their normal weight counterparts (44). The results of the DEI are as well in accordance with Powell et al. who showed the importance of family, friend and peer structures of weight development in the way of social contagion and the feeling of a sense of belonging and social support. This goes ahead with social engagement in groups and projects (41). Kouvonen et al. showed for older adults who participated in social activities a healthier BMI than for non-participants (75). Individuals who stated higher “food insecurity” seem to have higher overweight and obesity prevalence rates than individuals with a food security focus (76). Furthermore, it can be suggested that convenience food consumption is associated with being overweight or obesity and additionally, overweight people have less cooking skills than their normal weight counterparts, especially in men living alone (43).

Focusing on gender-specific aspects, it is shown that males tend to have more daily convenience behaviors than females and the total CBQ-Score seems to be reliable and is able to detect convenience behaviors in different population groups, independent of their physical activity level. Biswas et al. stated a prolonged sedentary time has an independent association with diseases like i.e., cardiovascular diseases, type 2 diabetes mellitus and cancer which is contributed by overweight and obesity regardless of physical activity (23). But physical inactivity itself, i.e., in term of “too much sitting” seems also linked to being overweight and obese (77, 78), even there are studies which found no relationship between sedentary time and weight outcomes (79). Interestingly, the ABI is a better independent predictor of overweight and obesity status than the well-established factor physical activity when measured with the HPAQ (45), and the HPA-Score did not remain in the final regression model. In contrast, Cheng and Furnham, found a significant and independent association between physical exercise and adult obesity (29). Therefore, it can be suggested that convenience behaviors play an important role in the development of overweight and that they have to be further pursued. In agreement with González et al. it can be concluded that being physically active is not enough and there is as well a need to avoid sedentary behavior (80).

Three component factor structures explained 43.4% of variance. This is comparable to the results of other validation studies. Both the present Cronbach's Alpha and the three-factor intern-reliability had an acceptable value (66, 81, 82).

Limitations of the study includes the use of self-reported weight and height to calculate BMI. The distribution of overweight and obese BMI groups in our sample is a little lower in comperance to the national and international data (83, 84). Participants tend to underestimate their body weight and to overestimate their body height, in particular the overweight and obese subjects (85). Thus, calculations of BMI may be biased and lead to misinterpretation of the found associations in this study between the convenience behavior and overweight and obesity status. Further studies should include direct measurements of weight and height. Additionally, other elements of the CBQ or the comparative questionnaire are subjective: i.e., screen time, physical activity. Although we observed a stable factor structure for the SIBI and the DEI, we found no significant association with overweight and obesity status. Underreporting of body weight and over-reporting of height could be one explanation. Therefore, additional or alternative questions may have to be developed for the SIBI and DEI to be appropriately used as well as a higher sample size has to be employed (especially in males) to address how socio-demographic variables affect the association between the convenience behavior and being overweight. It was attempted to include different population groups with different daily behavior patterns regarding work, leisure-time, and physical activity into the questionnaire. However, the considerations were based on a study with a suboptimal sample size and gender distribution. The majority of respondents were women with the exception of the “bus drivers.” In the group “parents from kindergartners and parents of school children,” men and women had an equal choice to answer the questionnaire, but mostly women responded to the questionnaire. Furthermore study recruitment strategies may have influenced the study outcomes because study participation was voluntary, and thus may probably lead to the fact that particular individuals with specific behavior patterns did not participate.

## Conclusion

The CBQ is a viable and reliable self-administered questionnaire to identify dimensions of convenience behaviors related to overweight and obesity. The factors of the CBQ have good to moderate test-retest reliability, and acceptable internal consistency. Further evaluation and validation are needed in longitudinal studies and additional populations. Beyond the established physical (in)activity and sedentary behaviors, the CBQ is a new and independent measurement tool of overweight and obesity status in adulthood. Interestingly, avoidance behavior shows a better relationship to overweight status than the physical activity status when measured with the HPAQ. In accordance with theoretical considerations and results of other studies, this issue raises the question whether convenience behavior has an effect on overweight and obesity status or has a moderation function. We therefore suggest researchers use the CBQ as additional measures in epidemiological studies for determining factors of overweight and obesity.

## Ethics Statement

The study was approved by the Institutional Review Board (IRB) of the city of Mainz which is responsible for evaluating the task of epidemiological research with personal data from participating subjects under ethical and legal aspects. Finally, the study was approved by the data protection commissioner according to the State Data Protection Act of Rhineland Palatinate which is required for study performance. The participants gave their written consent to participate in the survey. To increase the level of anonymity of the survey the collection of personal data was reduced to a minimum.

## Author Contributions

MD was involved in data analysis and wrote the final manuscript. SH was involved in study design, data collection, data analysis and drafted the manuscript. CB and DH were involved in study design, data collection, and data analysis. PS is the principal investigator of this study, contributed to the study design and was also involved in statistical data analysis and writing the manuscript.

### Conflict of Interest Statement

The authors declare that the research was conducted in the absence of any commercial or financial relationships that could be construed as a potential conflict of interest.
